# Relationship between Prostate Inflammation and Periodontal Disease—A Systematic Review and Meta-Analysis

**DOI:** 10.3390/jcm12186070

**Published:** 2023-09-20

**Authors:** Pablo Ortíz de Urbina Comerón, Álvaro Zubizarreta-Macho, Ana Belén Lobo Galindo, José María Montiel-Company, María-Fernanda Lorenzo-Gómez, Javier Flores Fraile

**Affiliations:** 1 Department of Surgery, Faculty of Medicine and Dentistry, University of Salamanca, 37008 Salamanca, Spain; pabloortizdeurbina@usal.es (P.O.d.U.C.); mflorenzogo@yahoo.es (M.-F.L.-G.); j.flores@usal.es (J.F.F.); 2 Department of Implant Surgery, Faculty of Health Sciences, Alfonso X el Sabio University, 28691 Madrid, Spain; alobogal@hotmail.com; 3 Department of Stomatology, Faculty of Medicine and Dentistry, University of Valencia, 46010 Valencia, Spain; jose.maria.montiel@uv.es; 4 Servicio de Urología del Hospital, Universitario de Salamanca, 37007 Salamanca, Spain

**Keywords:** periodontitis, prostatitis, prostate specific-antigen, odds ratio, hazard ratio

## Abstract

The aim of this systematic review and meta-analysis was to analyze the association between periodontal disease and prostate inflammation with a null hypothesis stating that periodontal disease does not increase the incidence of prostate inflammation. Materials and methods: A systematic literature review and meta-analysis of longitudinal observational cohort and case-control studies that evaluated the odds ratio or hazard ratio and confidence interval was undertaken based on the Preferred Reporting Items for Systematic Reviews and Meta-Analyses (PRISMA) recommendations (2020). A total of four databases were consulted in the literature search: PubMed-Medline, Scopus, Embase, and Web of Science. After eliminating duplicated articles and applying the inclusion criteria, seven articles were selected for the qualitative and quantitative analyses. Results: Four observational cohort studies and three observational cohort case-control studies were included in the meta-analysis. The four observational cohort studies were combined using the random effects model to estimate a hazard ratio of 1.32 with a confidence interval of 95% between 0.87 and 1.77. The meta-analysis presented high heterogeneity (Q test = 56.1; *p* value < 0.001; I^2^ = 94.9%). Moreover, the three observational case-control studies were combined using the random effects model to estimate an odds ratio of 1.62 with a confidence interval of 95% between 1.41 and 1.84. The meta-analysis presented high heterogeneity (Q test = 1.07; *p* value = 0.782; I^2^ = 0%). Conclusions: The incidence of periodontal disease does not increase the risk of the incidence of prostate inflammation.

## 1. Introduction

Previous studies have suggested that inflammation of the dental supporting tissues and inflammation of the prostate gland could share a common etiology and reciprocally influence their incidence. This would indicate that dentists can influence the development and establishment of prostate diseases, since by controlling periodontal disease, they can prevent the appearance of prostate diseases.

In addition, Barone et al. (2023) reported a bacterial etiology of 5–10% of all prostatic inflammation and highlighted bacterial reduction as the most efficacious treatment approach to treating prostatitis [1]. Furthermore, De Luca et al. (2020) reported that granulomatous prostatitis, which is considered an uncommon (3.3%) chronic prostate inflammation with an autoimmune etiology, has shown an association with psoriasis (another autoimmune disease). This fact could demonstrate that certain pathologies may have a common etiology that leads to their establishment and development [2].

Prostatic inflammation or prostatitis is considered one of the most common prostate pathologies, with a prevalence of 11% in people under 50 years of age [3]. In addition, it was described as a “pathology” by the National Institutes of Health (NIH) in 1995, considering its multifactorial etiology [4].

Inflammation of the prostate gland is a condition that must be taken into consideration since it can sometimes lead to the development of prostate cancer. Additionally, benign prostatic hyperplasia is a common condition of the prostate gland, which is usually diagnosed incidentally during digital rectal examination and is confirmed by ultrasound-guided transrectal biopsy, as well as by prostate-specific antigen (PSA) levels [5].

In recent years, the molecular characterization of oral microbiota has facilitated the detection of 700 bacterial species or biotypes with the capability of colonizing the tissues in the oral cavity. However, a healthy individual harbors between approximately 150 and 200 different bacterial species, of which between 10 and 30 can cause periodontal diseases (PD) [6]. Subgingival bacterial counts show that a healthy individual harbors 103 colony-forming units (CFUs), whereas individuals with established PD can harbor up to 108 CFUs [7]. These types of infections are responsible for damaging tooth support tissues [8,9,10].

Periodontal diseases are characterized by their high prevalence. Epidemiological studies indicate that between 5 and 20% of the population suffers from advanced forms of periodontitis [11,12]. The etiopathogenesis of periodontal disease is strongly associated with the formation of dental plaque. Dental plaque is a complex polymicrobial biofilm model, whose process initiates with the introduction, establishment, and growth of primary colonizing bacteria on teeth, such as *Streptococcus oralis*.

Longitudinal clinical trials have shown that adequate control of bacterial plaque can prevent periodontitis and that the withdrawal of oral hygiene mechanisms is accompanied by an increase in bacterial plaque and the onset of this disease [13,14,15].

Previous studies have suggested a possible relationship between periodontal pathology and prostate inflammation, since multiple typical oral pathogens of periodontitis—*Porphyromona gingivalis*, *Fusobacterium nucleatum*, *Actinomyces actinomycetemcomitans*, *Treponema denticola*, and *Escherichia coli*—have also been found in cultures extracted from prostate disorders [16]. Therefore, this fact highlights the relevance of oral health as an integral part of the general health and well-being of patients. Moreover, some theories have been developed to explain the relationship between periodontal inflammation and prostate disease, such as the distant migration of oral microbial agents via the hematogenous route. This hypothesis was verified by Estemalik et al. (2017), who carried out DNA tests using dental plaque and prostatic fluids, finding the presence of at least one bacterial biotype in both tests in 64.7% of samples. This finding further highlighted the critical role of microbial flora in human health and disease [17].

Additionally, another possibility could be an association with the presence of certain proinflammatory cytokines of periodontal origin, which would cause a state of chronic inflammatory weakness that could stimulate the appearance of systemic disorders. Some of these cytokines are interleukins (ILs) (IL-1β, IL-6 and IL-8; tumor necrosis factor-α (TNF-α); interferon-γ). The possible inflammatory affectation by cytokines could explain abacterial prostatitis.

Finally, another hypothesis theorizes that the inflammatory response of the prostate gland is caused by a local increase in PSA levels, which could occur distantly, even in the periodontium. The generation of PSA in the periodontium tissues would imply the systemic alteration of pro-inflammatory mediators, establishing a dysfunction of these mediators.

Therefore, previous longitudinal clinical trials have shown that adequate control of bacterial plaque can prevent periodontitis and secondarily prostate inflammation and that the withdrawal of oral hygiene mechanisms is accompanied by an increase in bacterial plaque and the onset of this disease [11,12,13]. Many authors emphasize the importance of oral hygiene techniques to prevent the formation of bacterial biofilm [18].

The aim and rationale of this systematic review and meta-analysis were to analyze and compare the association between the incidence of periodontal disease and the risk of increased incidence of prostate inflammation, with a null hypothesis (H_0_) stating that periodontal disease does not increase the incidence of prostate inflammation.

## 2. Materials and Methods

### 2.1. Study Design

This bibliographic search was conducted following PRISMA (Preferred Reporting Items for Systemic Reviews and Meta-Analyses; http://www.prisma-statement.org) guidelines for systematic reviews and meta-analyses (INPLASY registration number: INPLASY202350030; DOI number: 10.37766/inplasy2023.5.0030). The review also fulfilled the PRISMA 2020 Checklist [19].

### 2.2. Focused Question

The PECO (population, exposition, comparison, outcome) question was: ‘Do men exposed to periodontal disease have a higher risk of suffering prostate inflammation?’ with the following components: population: men affected by periodontal disease and prostate inflammation; exposition: periodontal disease and prostate inflammation; comparison: men not affected by periodontal disease and prostate inflammation; and outcomes: prostate inflammation.

### 2.3. Databases and Search Strategy

An electronic search was conducted in the following databases and gray literature: PubMed; Scopus; Embase; and Web of Sciences (A.Z.-M.; J.M.M.-C.). The search covered all the literature published internationally up to May 2023. The search included seven medical subject heading (MeSH) terms: ‘periodontitis’; ‘prostatitis’; ‘prostate-specific antigen’; ‘odds ratio’; and ‘hazard ratio’. The Boolean operators applied were (‘OR’ and ‘AND’). The search terms were structured as follows: ‘((periodontitis OR periodontal disease) AND (prostatitis OR prostate-specific antigen)) AND (odds ratio OR hazard ratio)’. Two researchers (A.Z.-M; J.F.F.) conducted the database searches in duplicate independently. Titles and abstracts were selected by applying inclusion and exclusion criteria.

### 2.4. Study Selection

Titles and abstracts were selected by two authors (A.B.L.G.; J.M.M.-C.), applying inclusion and exclusion criteria.

Inclusion criteria: longitudinal observational cohort and case-control studies. No restriction was placed on the year of publication or language.

Exclusion criteria: systematic reviews of the literature, clinical cases, case series with up to 5 patients and editorials; studies that include women or men under 18 years of age; and studies with samples of 5 or fewer patients. The following data were extracted from each article by two authors (P.O.d.U.C.; A.Z.-M.): author and year of publication; title and journal in which the article was published; sample size (n); follow-up time, odds ratio or hazard ratio; and confidence interval. Studies that analyzed the incidence risk of periodontal disease and prostate inflammation were included in the systematic review and meta-analysis.

### 2.5. Data Extraction and Study Outcomes

Data extraction was conducted in duplicate (P.O.d.U.C.; J.M.M.-C.) using predefined Excel spreadsheets and accounting for the following items: author and year, study type, sample size, follow-up in months, odds ratio or hazard ratio, and 95% confidence intervals.

### 2.6. Methodological Quality Assessment

The risk of bias in the studies selected for review was assessed by two authors (J.F.F.; P.O.d.U.C.) using the Newcastle–Ottawa scale for methodological quality assessment of longitudinal observational cohort and case-control studies. The Newcastle–Ottawa scale consists of three items that evaluate selection, comparisons, and results [20]. The level of agreement between evaluators was determined using Kappa scores.

### 2.7. Quantitative Synthesis—Meta-Analysis

The statistical data collection and analysis were conducted by two authors (A.Z.-M.; J.M.M.-C.). The studies included in the meta-analysis were combined using a random effects model with the maximum likelihood method used for effect size estimation as the odds ratio or hazard ratio. Heterogeneity between the combined studies was assessed using the Q test (*p*-value < 0.05) and was quantified using the I^2^. Heterogeneity was considered to be slight if it was between 25 and 50%, moderate between 50 and 75%, and high if > 75%. The existence of statistical significance was assessed using the Z test (*p*-value < 0.05). Meta-analyses were represented with forest plots. Publication bias was assessed using the trim-and-fill adjustment method and represented with funnel plots.

## 3. Results

### 3.1. Flow Diagram

**Figure 1:**
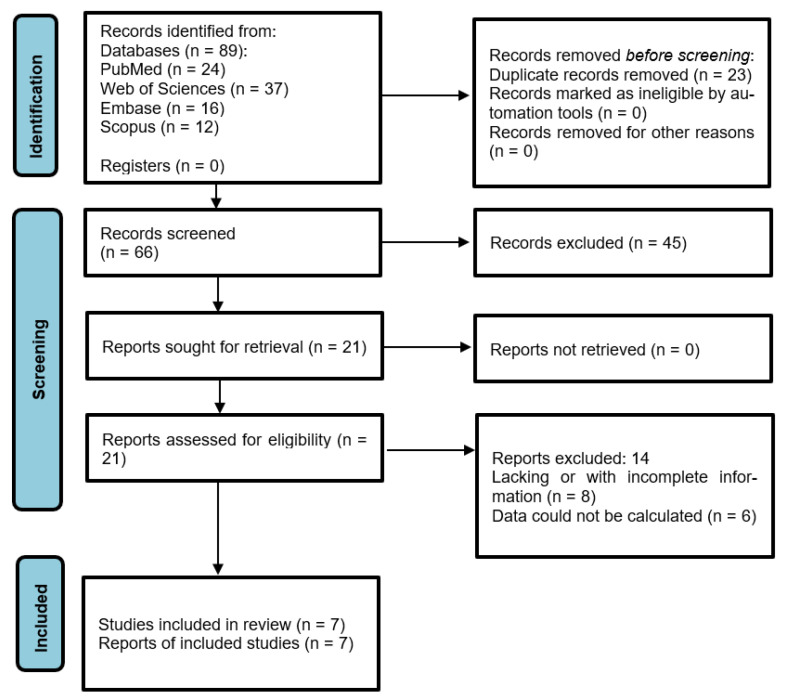
Preferred Reporting Items for Systematic Reviews and Meta-Analyses (PRISMA) flow diagram.

The initial electronic search identified 24 articles in PubMed, 37 in Web of Sciences, 16 in Embase, 12 in Scopus, and none in gray literature. Of the total 89 works, 26 were discarded as duplicates. After reading the titles and abstracts, a further 42 were eliminated leaving a total of 21. A further 14 were rejected as they failed to fulfill the following inclusion criteria: they did not include survival rate data, or they did not include a hazard ratio, odds ratio, or confidence interval. A final total of seven articles were included in the qualitative synthesis. Seven articles were included in the quantitative synthesis as these included all the data and variables required (Figure 1).

### 3.2. Qualitative Analysis

Of the seven articles included, four were longitudinal observational studies (cohort studies) [21,22,23,24] and three were cross-sectional observational clinical studies (case-control studies) [25,26,27] (Table 1).

### 3.3. Quality Assessment

The results of the methodological quality assessment were performed by one author (A.Z.-M.) using the Newcastle–Ottawa scale and are shown in Table 2 and Table 3. The Newcastle–Ottawa scale obtained scores between 6 and 8, indicating high methodological quality and low risk of bias.

### 3.4. Quantitative Analysis

#### 3.4.1. Hazard Ratio of Prostate Inflammation in Patients Affected by Periodontal Disease

**Figure 2:**
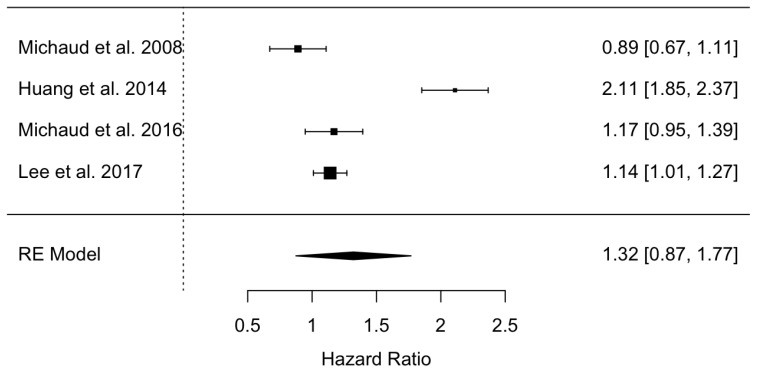
Forest plot of the hazard ratio meta-analysis of prostatitis in periodontal patients versus non-periodontal patients [21,22,23,24].

Four studies, in which only two showed a significant association, were combined using the random effects model and the maximum likelihood method to estimate a hazard ratio = 1.32 and with a 95% confidence interval between 0.87 and 1.77, indicating the absence of significance. Periodontal disease increases the risk of prostatitis by 1.32 times, although it is not significant. The meta-analysis presented high heterogeneity; a Q test = 56.1 with a *p* value < 0.001 and I^2^ = 94.9%. (Figure 2).

#### 3.4.2. Odds Ratio of Prostate Inflammation in Patients Affected by Periodontal Disease

**Figure 3:**
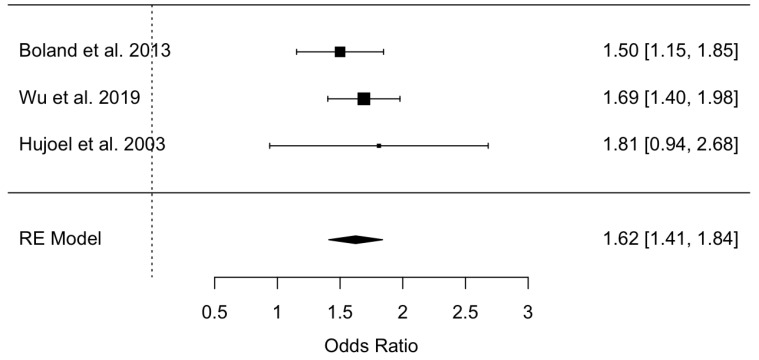
Forest plot of the odds ratio meta-analysis of prostatitis in periodontal patients versus non-periodontal patients [25,26,27].

Three studies were combined using the random effects model and the maximum likelihood method to estimate an odds ratio = 1.62 and with a 95% confidence interval between 1.41 and 1.84, indicating the existence of a statistical significance. The ratio between periodontal disease and healthy periodontal tissue is 1.6 times higher in patients with prostatitis. The meta-analysis did not present heterogeneity (Q test = 1.07; *p* value = 0.782; and I^2^ = 0%) (Figure 3).

### 3.5. Publication Bias

**Figure 4:**
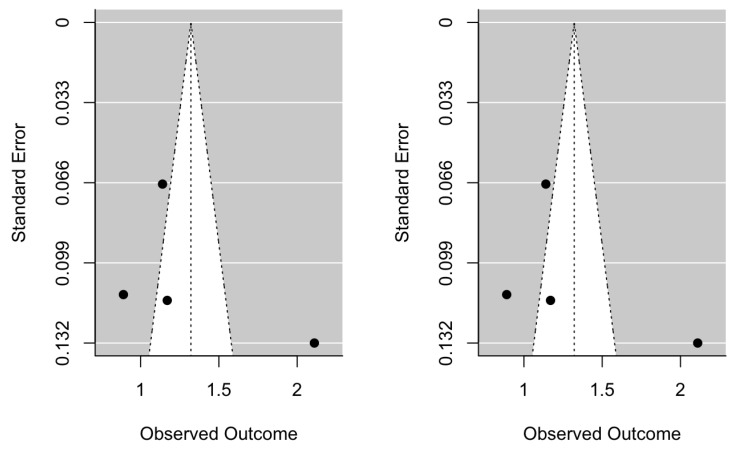
Initial hazard ratio funnel plot and after trim-and-fill adjustment.

**Figure 5:**
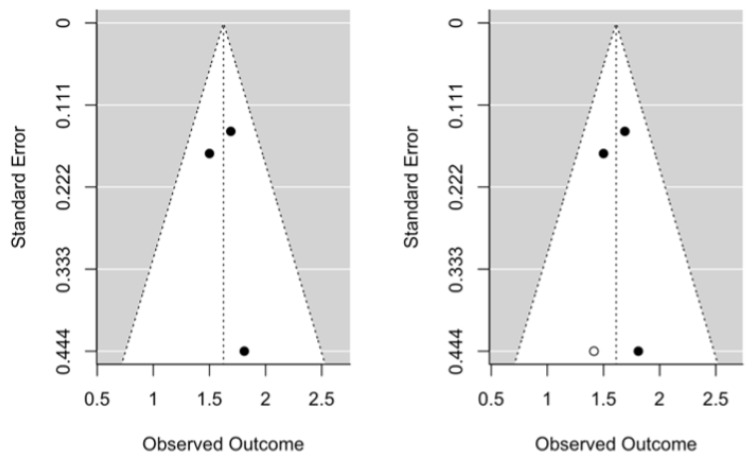
Initial odds ratio funnel plot and after trim-and-fill adjustment.

Publication bias was assessed using the Regression Test for Funnel Plot Asymmetry using a mixed-effects model, thus for the HR Test for Funnel Plot Asymmetry meta-analysis, z = 1.2326, *p* = 0.2177 (Figure 4), whereas for the OR meta-analysis Test for Funnel Plot Asymmetry, z = 0.2515, *p* = 0.8014 (Figure 5). Both results indicate the absence of publication bias.

## 4. Discussion

The results obtained in the present study accept the null hypothesis (H_0_) stating that periodontal disease does not increase the incidence of prostate inflammation.

Previous studies found a relationship between periodontitis and prostatitis [28,29], specifically in moderate-severe periodontitis, due to greater probing depths (LAC), worse plaque index (PI) values and worse bleeding rates, and higher prostate-specific antigen (PSA) levels (≥4 ng/mL), possibly due to oral microbiome biotypes (*Treponema denticola, P. gingivalis* and *Tanarella forsythea*). In addition, most of the patients (95%) with both pathologies have previously been affected by chronic bacterial prostatitis [30]. Therefore, Joshi et al. (2010) highlighted periodontal therapy as an adjunctive treatment for the control and management of chronic prostatitis. Non-surgical periodontal treatment consists of mechanical debridement of supra- and infra-gingival bacterial biofilm and subsequent instruction in oral hygiene maintenance techniques [31]. Furthermore, the clinical diagnosis of chronic prostatitis is characterized by difficulty urinating completely or painful urination, constant urinary incontinence, pain in the penis and/or testicles, and fever; therefore, emphasis should be placed on early diagnosis and the elimination of associated factors [32].

In addition, PD has also been linked to other systemic pathologies such as atherosclerotic valve disease, premature births, diabetes mellitus, chronic renal failure, and pulmonary infections [33]. Specifically, the oral cavity acts as a reservoir for microorganisms that may reach the prostate gland through metastatic infections, dissemination of bacterial toxins, and/or immune alterations, which can cause prostate damage once they cross the intraepithelial tissue of the prostate gland [34]. This migration could lead to the establishment of a new neoplastic transformation. Indeed, it would be associated with Gram-negative bacteria, the predominant etiological agents in periodontitis and in categories I and II of prostatitis.

Moreover, Liu et al. (2015) suggested that the presence of chronic prostatitis and periodontitis could inhibit self-healing and, therefore, maintain both diseases in the chronic inflammatory phase [35]. In addition, Endo M (2017) proposed avoiding bacterial reinfection by prescribing antibiotic treatment for 15 days to eliminate the residual bacterial load from the original infection [36]. Most of the authors agreed with applying a non-surgical periodontal treatment to achieve an improvement in PSA levels, especially in patients affected with a moderate-severe prostate pathology. However, the reduction in PSA levels does not exempt us from performing prostate biopsies that could report other prostate alterations [37].

Although most studies highlighted the beneficial effect of periodontal treatment on the reduction of PSA levels, Kruck et al. (2017) showed that supra- and infra-gingival biofilm debridement alongside adequate instruction in oral hygiene techniques was not enough to significantly reduce PSA levels, probably due to the limited number of PSA measurements and the prostatitis classification of the selected patients [38]. However, Alwithanami et al. (2015) concluded that periodontal treatment for 4–8 weeks led to a significant reduction in PSA levels, compared with the control group; specifically, 21 out of 27 patients experienced a significant decrease in PSA levels [39]. Likewise, Fang et al. (2021) performed a randomized clinical trial and did not find an association between the oral microbiome and prostate changes; however, the manipulation of the microbial composition could effectively prevent the establishment of periodontal and systemic pathology [40]. Alwithanami et al. (2015) also reported that it may not only be the disease that is involved in the deterioration of the state of the prostate gland since the loss of teeth would also be a relevant factor in the development of prostatitis and the subsequent development of prostate cancer. For all these reasons, the establishment of periodontal therapy was suggested as a required treatment to improve dental health and reduce high PSA levels [37]. However, periodontal therapy with oral hygiene instructions is not considered an ideal treatment choice for all studies, since—as demonstrated in those patients with abacterial prostatitis—it may not have plausible effects at the PSA level. In addition, secondary bacterial infections could demonstrate chronic maintenance of asymptomatic prostatitis through chronic pelvic pain syndrome [41].

Additionally, Fu et al. (2021) stated that diabetes mellitus could lead to the development of gingivitis and subsequent periodontitis, which would likely become chronic. The prostate could consequently be affected, leading to prostatitis and benign prostatic hyperplasia and/or prostate cancer. Many risk and environmental factors have been associated; however, the development of prostatitis in patients with periodontitis is up to 4.6 times more likely than in patients without periodontitis [39].

Therefore, it is essential to highlight periodontal treatment, specifically in patients with unfavorable LAC, PI, and gingival index values [29]. Since the oral cavity constitutes a microbial reservoir for Gram-positive and Gram-negative bacteria, it is possible that the reduction of the bacterial load would consequently produce a distant reduction of multiple Gram-negative bacteria which could reduce the high levels of PSA and therefore the concomitant immune and inflammatory responses that could be present locally or systemically.

The relationship between periodontitis and prostatitis has been widely described in previous studies. Additionally, various studies have highlighted the incidence of periodontal disease with other systemic diseases such as cardiovascular diseases, acute myocardial infarctions, diabetes mellitus, adverse effects of pregnancy, respiratory disorders, osteoporosis, obesity, malnutrition, rheumatoid arthritis, and a wide variety of cancers.

However, the scant published scientific evidence is not enough to define in greater depth the advantages and disadvantages of periodontal therapy in patients with chronic prostatitis. Therefore, it is quite pertinent that further randomized clinical trials are carried out to confirm the association of periodontal disease with chronic prostatitis.

The authors report that the reduced number of articles included in this systematic review and meta-analysis could be considered a limitation; therefore, we encourage researchers to perform further and better-designed clinical studies with higher quality.

## 5. Conclusions

Meta-evidence suggests that the incidence of periodontal disease does not increase the risk of incidence of prostate inflammation; however, more clinical studies are necessary to confirm this statement.

## Figures and Tables

**Table 1:** Qualitative analysis of articles included in the systematic review.

Author/Year	Study Design	N_Control Group	HN_Control Group	IC_Control Group
Michaud et al., 2008 [21]	Cohort	48,275	0.89	0.71–1.10
Huang et al., 2019 [23]	Cohort	38,092	2.11	1.63–2.73
Michaud et al., 2016 [22]	Cohort	19,933	1.17	0.94–1.47
Lee et al., 2017 [24]	Cohort	1235	1.5	1.05–2.10
Wu et al., 2019 [25]	Case-control	2171	1.81	0.76–4.34
Hujoel et al., 2003 [27]	Case-control	5240	0.49	0.19–1.26
Boland et al., 2013 [26]	Case-control	1240	1.5	1.05–2.10

**Table 2:** Assessment of methodological quality of observational cohort studies, according to the Newcastle–Ottawa scale.

Author/Year	Selection				Comparability			Outcome		
	Representative of the Exposed Cohort	Selection of External Control	Ascertainment of Exposure	Outcome of Interest Not Present at the Start of the Study	Main Factor	Additional Factor	Assessment of Outcomes	Sufficient Follow-Up Time	Adequacy of Follow-Up	Total
Michaud et al., 2008 [21]	*		*	*			*	*	*	6/9
Huang et al., 2019 [23]	*	*	*	*	*		*	*	*	8/9
Michaud et al., 2016 [22]	*		*	*			*	*	*	6/9
Lee et al., 2017 [24]	*		*	*			*	*	*	6/9

*: Means that the response is affirmative.

**Table 3:** Assessment of methodological quality of observational case-control studies, according to the Newcastle–Ottawa scale.

Author/Year	Selection				Comparability			Outcome		
	Representative of the Exposed Cohort	Selection of External Control	Ascertainment of Exposure	Outcome of Interest Not Present at the Start of the Study	Main Factor	Additional Factor	Assessment of Outcomes	Sufficient Follow-Up Time	Adequacy of Follow-Up	Total
Boland et al., 2013 [26]	*		*	*			*	*	*	6/9
Wu et al., 2019 [25]	*		*	*			*	*	*	6/9
Hujoel et al., 2003 [27]	*		*	*			*	*	*	6/9

*: Means that the response is affirmative.

## Data Availability

Data available on request due to restrictions, e.g., privacy or ethical.
